# Microvascular decompression and aneurysm clipping for a patient with hemifacial spasm and ipsilateral labyrinthine artery aneurysm: A rare case report and literature review

**DOI:** 10.1111/cns.13783

**Published:** 2021-12-24

**Authors:** Yufei Liu, Fanfan Chen, Zongyang Li, Jihu Yang, Xiejun Zhang, Lei Chen, Liwei Zhang, Guodong Huang

**Affiliations:** ^1^ Neurosurgical Department The First Affiliated Hospital of Shenzhen University Shenzhen Second People’s Hospital Shenzhen China; ^2^ Neurosurgical Department Beijing Tiantan Hospital Capital Medical University Beijing China

**Keywords:** aneurysm, case report, hemifacial spasm, microvascular decompression

## Abstract

Preoperative MRI results showed a vascular anomaly at the REZ of the left facial nerve (Figure A) and an anomaly at the internal auditory canal (Figure B). The left AICA was identified as the offending vessel compressing the left facial nerve at the REZ (Figure C). After the artery was dissociated and Teflon felt was placed between the involved vessel and the facial nerve (Figure D), electrophysiological monitoring indicated that the AMR had disappeared (Figure E).

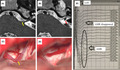

## CONFLICT OF INTEREST

The authors declare no conflict of interest.

## CONSENT FOR PUBLICATION

Written informed consent was obtained from the patient for publication of this case report and any accompanying images. A copy of the written consent is available for review by the editor of this journal. All authors agreed to publication.

Dear Editor:

Hemifacial spasm (HFS) is characterized by involuntary, unilateral, brief, and persistent paroxysmal contractions of the facial muscles innervated by the facial nerve. The most common etiology of primary HFS reported in the literature is an offending artery, which compresses the facial nerve at the root entry/exit zone.^1^ A labyrinthine artery (LA) aneurysm mostly distal to the anterior inferior cerebellar artery (AICA) is rare. To the best of our knowledge, there has been no previous report about the phenomenon of HFS with an ipsilateral LA aneurysm. We describe this phenomenon in one of our patients and discuss its possible cause and treatment.

**FIGURE 1:**
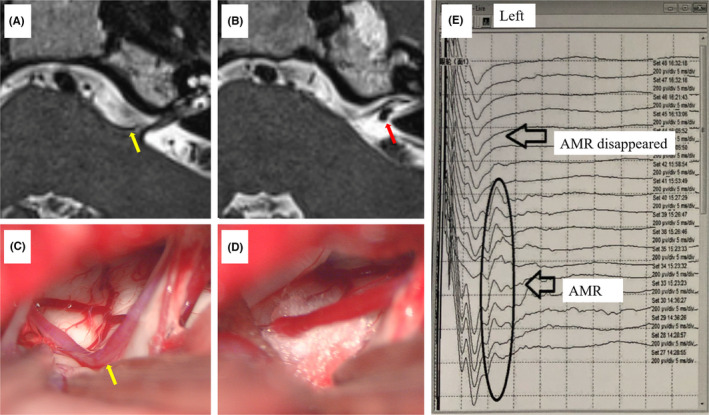
MRI showing the offending vessel at the root entry/exit zone (A) and the small aneurysm of the LA (B). The offending vessel during surgery (C). Teflon felt was placed between involved vessel and facial nerve (D). The AMR disappeared (E). AMR, abnormal muscle reaction; LA, labyrinthine artery; MRI, magnetic resonance imaging

A 51‐year‐old woman complaining of involuntary left HFS for 5 years was admitted to the hospital on August 31, 2020. Her past medical history, family history, and social history were not significant. Except for left HFS, her neurological examination was normal. Preoperative magnetic resonance imaging results showed a vascular anomaly at the root exit zone of the left facial nerve (Figure 1A) and an anomaly at the internal auditory canal (Figure 1B). Preoperative electrophysiological examination revealed abnormal muscle reaction (AMR) on the left side.

**FIGURE 2:**
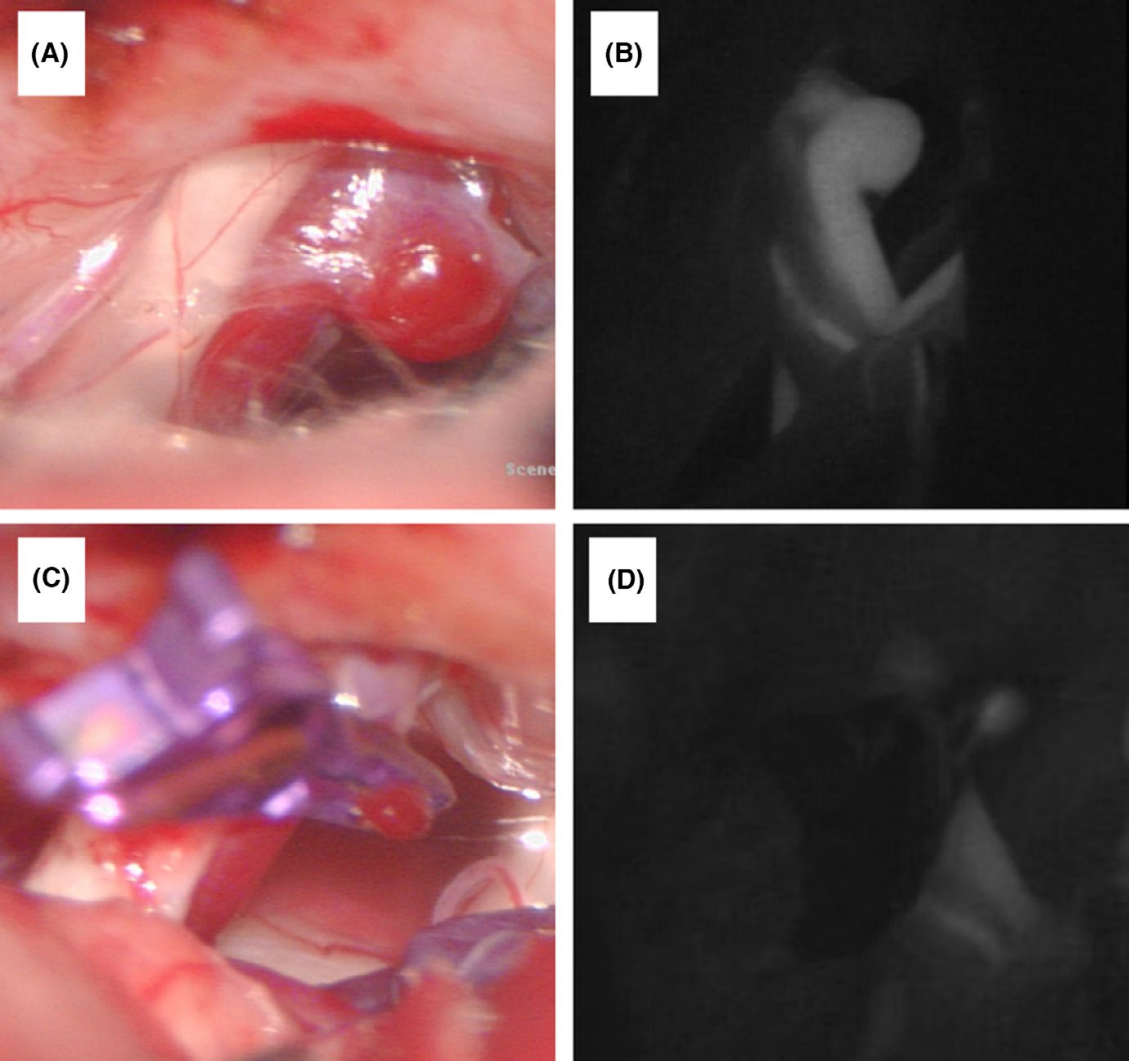
Small aneurysm of the LA was identified by ICG FA (A, B). Aneurysmal neck clipping (C). Complete clipping of the aneurysmal neck and vessel patency was evaluated by ICG FA (D). ICG FA, indocyanine green fluorescence angiography; LA, labyrinthine artery

On September 3, 2020, a left retrosigmoid craniectomy was performed with the patient in the contralateral lateral decubitus position under general endotracheal anesthesia. First, a small aneurysm of the LA was found by indocyanine green fluorescence angiography (ICG FA) at the opening of the internal auditory canal (Figure 2A,B). Sharp separation and dissociation of the arachnoid between the parallel LA and the facial and acoustic nerves were performed, but the AMR did not disappear. Second, the left AICA was identified as the offending vessel compressing the left facial nerve at the root entry/exit zone (Figure 1C). After the artery was dissociated and Teflon felt was placed between the involved vessel and the facial nerve (Figure 1D), electrophysiological monitoring indicated that the AMR had disappeared (Figure 1E). Finally, a straight miniature titanium aneurysm clip was placed at the aneurysmal neck, and satisfactory clipping was confirmed by ICG FA (Figure 2C,D).

Her left HFS disappeared after surgery. She was discharged from the hospital with left mild hearing loss 7 days after the surgery. Postoperative head computed tomography showed no cerebral hemorrhage or cerebral infarction. She refused a postoperative digital subtraction angiography (DSA) control. One year after the operation, the patient had had no recurrence of HFS, her left‐side hearing had recovered, and she had resumed work.

The long‐term curative effect rate of microvascular decompression for HFS is high, and complications are uncommon and usually transient.^2^ It has been reported that microvascular decompression could be a safe and effective treatment for patients with HFS.^2^ Microvascular decompression using a gelatin sponge with a FuAiLe medical adhesive was considered to be a modified effective technique for patient with HFS involving the vertebral artery.^3^ Reports have suggested that AMR monitoring provides valuable information during the microvascular decompression operation for HFS.^4^ Here, the LA and facial auditory nerve were found to be parallel and closely related intraoperatively. After sharp separation and dissociation of the arachnoid between the parallel LA and its aneurysm and the facial/acoustic nerves, the AMR did not disappear, which indicated that the LA with its aneurysm was not the offending vessel. Rather, the AICA at the root entry/exit zone was the offending artery, and when it was separated and padded away, the AMR disappeared immediately.

The LA usually arises from the meatal loop of the AICA (90%) or basilar artery (10%).^5^ The loop is extrameatal to the internal auditory canal in 30%, at the opening of the internal auditory canal in 20%, or intrameatal in 35%.^5^ In the present case, the small aneurysm of the LA was at the opening of the internal auditory canal. Previous articles have reported ruptured aneurysms distal or intrameatal to the AICA.^6^, ^7^. After extensive discussion with and informed consent from the patient's family, we successfully clipped the aneurysmal neck. ICG FA is now widely used for the intraoperative assessment of vessel patency.^8^ Satisfactory clipping was confirmed by ICG FA again to ensure complete clipping of the aneurysmal neck and patency of the LA.

Postoperatively, the patient's HFS disappeared, but hearing loss occurred. The prevalence of hearing loss in HFS has been 13.39% in 23 studies with consistent perioperative audiograms.^9^ Whether ruptured intrameatal AICA aneurysms are clipped or embolized, most patients have postoperative facial nerve palsy and/or hearing deficit/loss.^7^ Kikkawa et al.^10^ believed that internal auditory artery occlusion was a potential cause of the loss of auditory and vestibular functions in a patient with a ruptured intrameatal AICA aneurysm. One year after the surgery, the present patient's hearing was restored. Postoperative DNA may be useful for such patients.

To summarize, surgery is an option for patients with HFS and ipsilateral internal auditory artery aneurysm. The etiology of this HFS was the vascular compression at the facial nerve entry zone, which was not related to the aneurysm. To treat a small LA aneurysm, aneurysm neck clipping with a miniature aneurysm clip is an option. ICG FA can be used for the intraoperative assessment of vessel patency and clipping of the aneurysmal neck.

## Data Availability

Data sharing not applicable to this article as no datasets were generated or analysed during the current study.
